# Predictors for surgical site infection in patients undergoing therapeutic or prophylactic intra-abdominal onlay mesh (IPOM) implantation in clean and contaminated surgical fields

**DOI:** 10.1007/s00464-023-10144-4

**Published:** 2023-06-13

**Authors:** Manuel O. Jakob, Adriana Brüggemann, Nina Moser, Daniel Candinas, Guido Beldi, Tobias Haltmeier

**Affiliations:** 1 Department of Visceral Surgery and Medicine, Inselspital, Bern University Hospital, University of Bern, Bern, Switzerland; 2 Institute of Microbiology, Infectious Diseases and Immunology (I-MIDI), Charité - Universitätsmedizin Berlin, Corporate Member of Freie Universität Berlin and Humboldt-Universität zu Berlin, Berlin, Germany

**Keywords:** Intra-abdominal onlay mesh, Surgical site infection, Risk factors, Hernia surgery, Abdominal surgery, Emergency surgery, Contamination, Infection

## Abstract

**Background:**

Prophylactic intra-abdominal onlay mesh (IPOM) implantation has been shown to reduce the rate of fascial dehiscence and incisional hernia. However, surgical site infection (SSI) in presence of an IPOM remains a concern. The aim of this study was to assess predictors for SSI following IPOM placement in hernia and non-hernia abdominal surgery in clean and contaminated surgical fields.

**Methods:**

Observational study including patients undergoing IPOM placement at a Swiss tertiary care hospital 2007–2016. IPOM implantation was performed in hernia and non-hernia elective and emergency abdominal surgery, including contaminated and infected surgical fields. The incidence of SSI was prospectively assessed by Swissnoso according to CDC criteria. The effect of disease- and procedure-related factors on SSI was assessed in multivariable regression analysis, adjusting for patient-related factors.

**Results:**

A total of 1072 IPOM implantations were performed. Laparoscopy was performed in 415 patients (38.7%), laparotomy in 657 patients (61.3%). SSI occurred in 172 patients (16.0%). Superficial, deep, and organ space SSI were found in 77 (7.2%), 26 (2.4%), and 69 (6.4%) patients, respectively. Multivariable analysis revealed emergency hospitalization (OR 1.787, p = 0.006), previous laparotomy (1.745, p = 0.029), duration of operation (OR 1.193, p < 0.001), laparotomy (OR 6.167, p < 0.001), bariatric (OR 4.641, p < 0.001), colorectal (OR 1.941, p = 0.001), and emergency (OR 2.510, p < 0.001) surgery, wound class ≥ 3 (OR 3.878, p < 0.001), and non-polypropylene mesh (OR 1.818, p = 0.003) as independent predictors for SSI. Hernia surgery was independently associated with a lower risk for SSI (OR 0.165, p < 0.001).

**Conclusion:**

This study revealed emergency hospitalization, previous laparotomy, duration of operation, laparotomy, as well as bariatric, colorectal, and emergency surgery, abdominal contamination or infection, and usage of non-polypropylene mesh as independent predictors for SSI. In contrast, hernia surgery was associated with a lower risk for SSI. The knowledge of these predictors will help to balance benefits of IPOM implantation against the risk for SSI.

**Graphical abstract:**

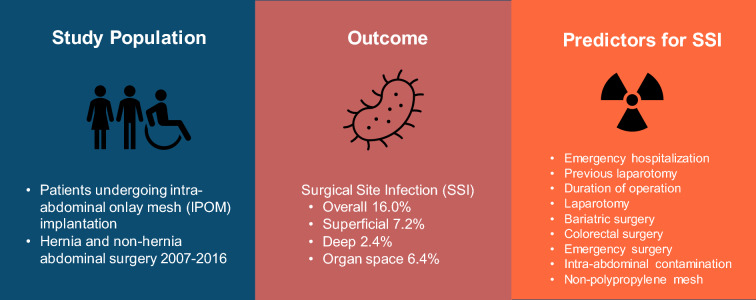

Intra-abdominal onlay mesh (IPOM) implantation for the repair of abdominal wall hernias has been shown to reduce the rate of recurrences compared to suture repair [1, 2]. Similarly, recent evidence suggests that prophylactic mesh implantation is superior compared to suture closure of the abdominal wall in patients with a high risk for incisional hernias [3–6]. As previously reported by our study group, prophylactic placement of synthetic IPOM is also feasible in patients with intraperitoneal contamination and leads to a decreased rate of incisional hernias in the long term [7]. Furthermore, we have shown that implantation of an IPOM in patients with fascial dehiscence and in open abdomen treatment leads to a decreased rate of revisional surgery, duration of hospital and intensive care unit stay, as well as a reduction of incisional hernias [4, 5].

On the other hand, mesh infections are a severe complication after mesh implantation and associated with hospital re-admission, re-operations, and hernia recurrences, resulting in high healthcare resource utilization [8]. Although mesh infections became less frequent over time, an incidence of mesh infections of one to eight percent has been reported after incisional or ventral hernia repair [9, 10]. There is currently no uniformly used definition of mesh infections available. The definition of mesh infection varied in previous studies and included the centers of disease control and prevention (CDC) criteria [11] for SSI, identification of pathogens after aspiration of a periprosthetic fluid collections, infections necessitating mesh removal, individualized criteria, or no specific definition [10, 12]. Even though the majority of SSI in presence of an IPOM can be treated locally without the need for revision surgery, SSI may require explantation of the foreign material [4, 13]. In these cases, source control can be difficult and the therapeutic regimen for such scenarios incompletely defined.

Taking into account the above-mentioned benefits for abdominal wall reinforcement and the potentially severe consequences of SSI in presence of a mesh, surgeons will have to stratify the risk for SSI when considering mesh implantation. Previous studies have reported risk factors for mesh infection after hernia surgery. In these studies, patient age, obesity, the American Society of Anesthesiologists (ASA) Physical Status Classification System score, smoking, diabetes mellitus, operative time, emergency setting, and the mesh position were described as risk factors for mesh infection [10, 12, 14].

However, IPOM is also used in patients undergoing abdominal surgery for other indications than incisional or ventral hernias, i.e., as a prophylactic measure or concomitant hernia repair in elective and emergency abdominal surgery, including patients with intra-abdominal contamination or infection. Therefore, the current study aimed to identify disease- and procedure-related predictors for SSI in patients undergoing IPOM placement, both, in hernia and non-hernia emergency and elective abdominal surgery.

## Materials and methods

### Study design

This is a retrospective single-center study performed at the Inselspital, Bern University Hospital, Switzerland. The study investigated predictors for SSI in patients undergoing laparoscopic or open abdominal surgery with placement of an IPOM. The study was approved by the ethics committee of the Canton of Bern, Switzerland (KEK-No. 217-00979).

Patients undergoing elective or emergency abdominal surgery and placement of an IPOM between January 2007 and December 2016 were included. In patients who underwent multiple IPOM implantations during the same hospital stay, only the first IPOM placement was included as index procedure. The excision and replacement of an IPOM in patients with SSI was counted as mesh removal.

IPOM implantation was performed using synthetic non-absorbable meshes for abdominal wall hernias, or as a prophylactic measure or concomitant hernia repair in emergency and elective abdominal surgery. IPOM was also utilized in patients with postoperative fascial dehiscence and patients undergoing open abdomen treatment, including cases with abdominal contamination.

Due to the known significantly lower rate of SSI in patients undergoing laparoscopy compared to laparotomy [15–19], patients included in the current study were further divided into a laparoscopy and laparotomy subgroup.

SSI was *prospectively* evaluated by the Swiss National Center for Infection Control (Swissnoso) [20] at 30 days postoperatively and defined according to CDC criteria [21]. The wound class was defined according to CDC criteria [21] as grade 1 for clean wounds, grade 2 for clean-contaminated wounds, grade 3 for contaminated wounds, and grade 4 for dirty-infected wounds.

Patient and treatment characteristics, as well as clinical outcomes were extracted from electronic medical records.

### Surgical technique for intraperitoneal onlay mesh implantation

The mesh was fixed on the abdominal wall with non-absorbable single stiches and absorbable tackers in laparoscopic surgery and non-absorbable running sutures in open surgery, respectively. In open surgery, abdominal wall closure was performed using an absorbable running suture in a standardized technique using a suture length to wound length ratio of 4:1. The skin was closed using single stiches or left open with negative pressure wound therapy. Negative pressure wound therapy was applied at the discretion of the attending surgeon in patients with an estimated high risk for SSI. Vacuum dressings were changed every three to four days and removed with secondary skin closure when there was evidence of granulation tissue and clean wound conditions. In laparoscopic surgery, fascial closure with absorbable figure of eight stiches was performed for 12 mm trocar incisions. The skin was closed with non-absorbable single stiches, skin staples, or absorbable intracutaneous sutures. Surgery was performed by different surgical teams, including attending surgeons, surgical fellows, and residents.

### Statistical analysis

Categorical variables were reported as numbers and percentages. The normality of distribution of continuous variables was assessed using the Shapiro–Wilk test. Continuous variables were reported as median and interquartile ranges (IQR).

Included patients were divided into two groups based on the presence of absence of an SSI, i.e., an SSI and non-SSI group.

Missing data were addressed using multiple imputation. A total of 20 itinerations were generated. Both, univariable and multivariable regression analysis were performed using the imputed dataset.

In univariable analysis, the effect of disease- and procedure-related factors on SSI was assessed using univariable logistic regression analysis.

In order to adjust the effect of individual disease- and procedure-related factors on SSI, multivariable analysis adjusting for patient-related factors was performed. Variables were adjusted for sex, age, BMI and ASA score in multivariable logistic regression analysis. Results of the regression analysis were reported as odds ratios (OR) with 95% confidence intervals (CI) and corresponding p-values.

**Fig. 1:**
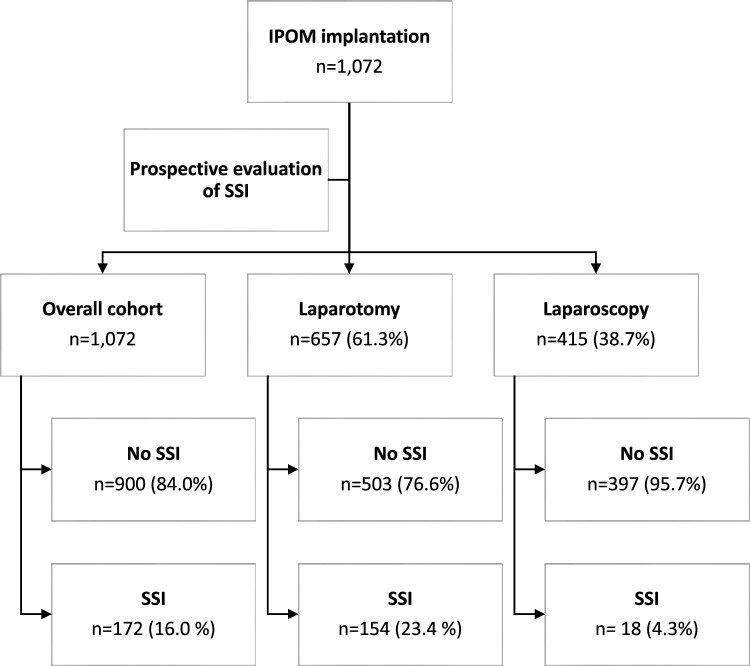
Patients undergoing intraoperitoneal onlay mesh implantation in hernia and non-hernia abdominal surgery 2007–2016. IPOM: intraperitoneal only mesh, SSI: surgical site infection

The described statistical analysis was performed in all patients included (overall cohort), as well as the laparoscopy and laparotomy subgroup (Fig. 1). A two-sided p-value of < 0.05 was considered statistically significant. Statistical analysis was performed using SPSS Statistics (IBM Corporation, Armonk, NY, USA).

## Results

### Patient characteristics

A total of 1072 patients undergoing abdominal surgery with placement of an IPOM were included in the current study. The majority of included patients were male (n = 649, 60.5%). Median age was 61.0 years (IQR 18.0) and median BMI 27.8 (IQR 8.0). Comorbid conditions were frequent with an ASA score ≥ 3 in 66.2% of the patients included (Table 1).

**Table 1:** Baseline characteristics

	Overall	Laparotomy	Laparoscopy
	(n = 1072)	(n = 657)	(n = 415)
Male sex	649/1072 (60.5)	395/657 (60.1)	254/415 (61.2)
Age (years)^a^	61.0 (18.0)	62.0 (17.0)	60.0 (18.0)
BMI (kg/m2)^a^	27.8 (8.0)	27.1 (7.6)	28.7 (8.1)
BMI > 30 kg/m2	297/880 (33.8)	176/545 (32.3)	121/335 (36.1)
Current smoker	230/621 (37.0)	140/374 (37.4)	90/247 (36.4)
Tumor	426/1062 (40.1)	313/651 (48.1)	113/411 (27.5)
Diabetes mellitus	188/1067 (17.6)	120/654 (18.3)	68/413 (16.5)
Arterial hypertension	488/1064 (45.9)	322/650 (49.5)	166/414 (40.1)
Heart disease	360/1065 (33.8)	236/651 (36.3)	124/414 (30.0)
Pulmonary disease	302/1066 (28.3)	214/652 (32.8)	88/414 (21.3)
Liver disease	242/1063 (22.8)	158/651 (24.3)	84/412 (20.4)
Kidney disease	276/1064 (25.9)	181/652 (27.8)	95/412 (23.1)
Anticoagulants	369/1033 (35.7)	237/629 (37.7)	132/404 (32.7)
Platelet aggregation inhibitors	217/1072 (20.2)	126/657 (19.2)	91/415 (21.9)
Immunosuppression	172/1033 (16.7)	95/627 (15.2)	77/406 (19.0)
ASA score			
1	73/921 (7.9)	10/536 (1.9)	63/385 (16.4)
2	238/921 (25.8)	96/536 (17.9)	142/385 (36.9)
3	436/921 (47.3)	289/536 (53.9)	147/385 (38.2)
4	166/921 (18.0)	133/536 (24.8)	33/385 (8.6)
5	8/921 (0.9)	8/536 (1.5)	–
ASA score ≥ 3	610/921 (66.2)	430/536 (80.2)	180/385 (46.8)
Emergency hospitalization	231/1065 (21.7)	176/654 (26.9)	55/411 (13.4)
Previous laparotomy	576/748 (77.0)	375/436 (86.0)	201/312 (64.4)
Duration of operation (hours)^a^	2.3 (2.0)	2.8 (2.2)	2.0 (1.3)
Laparotomy	657/1072 (61.3)	–	–
Type of surgery			
Upper-GI surgery	34/1072 (3.2)	31/657 (4.7)	3/415 (0.7)
Bariatric surgery	41/1072 (3.8)	32/657 (4.9)	9/415 (2.2)
Hernia surgery	532/1072 (49.6)	198/657 (30.1)	334/415 (80.5)
Hepato-biliary surgery	67/1072 (6.3)	55/657 (8.4)	12/415 (2.9)
Pancreatic surgery	22/1072 (2.1)	21/657 (3.2)	1/415 (0.2)
Colorectal surgery	205/1072 (19.1)	185/657 (28.2)	20/415 (4.8)
Acute Care Surgery	124/1072(11.6)	92/657 (14.0)	32/415 (7.7)
Open abdomen treatment	23/1072 (2.1)	23/657 (3.5)	–
Wound class			
1	522/1072 (48.7)	192/657 (29.2)	330/415 (79.5)
2	303/1072 (28.3)	241/657 (36.7)	62/415 (14.9)
3	80/1072 (7.5)	70/657 (10.7)	10/415 (2.4)
4	167/1072 (15.6)	154/657 (23.4)	13/415 (3.1)
Wound class ≥ 3	247/1072 (23.0)	224/657 (34.1)	23/415 (5.5)
Non-polypropylene mesh	277/1014 (27.3)	198/624 (31.7)	79/390 (20.3)

Values are numbers (percentages) unless indicated otherwise

^a^Values are medians (interquartile ranges)

*ASA* American society of anesthesiologists physical status classification system, *ICU* Intensive care unit

### Surgical characteristics

A total of 415 patients (38.7%) were operated by laparoscopy and 657 patients (61.3%) by laparotomy, respectively. Hernia surgery was performed in 532 patients (49.6%). In the remaining 540 patients (50.4%), IPOM was performed prophylactically or as a concomitant hernia repair in colorectal (n = 205, 19.1%), emergency (n = 124, 11.6%), hepato-biliary (n = 67, 6.3%), bariatric (n = 41, 3.8%), and upper gastrointestinal (n = 34, 3.2%) surgery. Open abdomen treatment was performed in 23 patients (2.1%).

A wound class ≥ 3, i.e., contaminated or dirty-infected wound, was found in 247 patients (23.0%). Non-polypropylene meshes were implanted in 277/1014 patients (27.3%); 79/390 patients (20.3%) in the laparoscopy subgroup and 198/624 patients (31.7%) in the laparotomy subgroup. (Table 1, Fig. 1).

### Surgical site infections

SSI and other clinical outcomes are outlined in Table 2. SSI occurred in 172 patients (16.0%). Of these, superficial, deep, and organ space SSI were found in 77 (7.2%), 26 (2.4%), and 69 (6.4%) patients, respectively. In the laparoscopy subgroup, SSI occurred in 18 patients (4.3%): 6 patients (1.4%) with superficial, three patients (0.7%) with deep, and nine patients (2.2%) with organ space SSI. In the laparotomy subgroup, 154 patients (23.4%) developed SSI. Of these, 71 (10.8%) were classified as superficial, 23 (3.5%) as deep, and 60 (9.1%) as organ space SSI.

**Table 2:** Clinical outcomes

	Overall	Laparotomy	Laparoscopy
	(n = 1072)	(n = 657)	(n = 415)
SSI overall	172/1072 (16.0)	154/657 (23.4)	18/415 (4.3)
SSI superficial	77/172 (44.8)	71/154 (46.1)	6/18 (33.3)
SSI deep	26/172 (15.1)	23/154 (14.9)	3/18 (16.7)
SSI organ space	69/172 (40.1)	60/154 (39)	9/18 (50.0)
Mesh removal	60/1072 (5.6)	53/657 (8.1)	7/415 (1.7)
Partial mesh removal	18/1072 (1.7)	18/657 (2.7)	0/415 (0.0)
Complete mesh removal	42/1072 (3.9)	35/657 (5.3)	7/415 (1.7)
Mesh removal by surgery type			
Upper-GI surgery	0/34 (0.0)	0/657 (0.0)	0/415 (0.0)
Bariatric surgery	6/41 (14.6)	6/32 (18.8)	0/9 (0.0)
Hernia surgery	12/532 (2.3)	8/198 (4.0)	4/334 (1.2)
Hepato-biliary surgery	5/67 (7.5)	5/53 (9.1)	0/12 (0.0)
Pancreatic surgery	3/22 (13.6)	3/21 (14.3)	0/1 (0.0)
Colorectal surgery	18/205 (8.8)	18/185 (9.7)	0/20 (0.0)
Acute Care Surgery	13/124 (10.5)	10/92 (10.9)	3/32 (9.4)
Open abdomen treatment	3/23 (13.0)	3/23 (13.0)	–
Sepsis	53/986 (5.4)	50/600 (8.3)	3/386 (0.8)
ICU admission	202/1072 (18.8)	184/657 (28.0)	18/415 (4.3)
Hospital length of stay (days)^a^	9.0 (14)	14.0 (20)	6.0 (4)

Values are numbers (percentages) unless indicated otherwise

^a^Values are medians (interquartile ranges)

*SSI* Surgical site infection, *ICU* Intensive care unit

### Mesh removal

Partial or total mesh removal due to SSI was required in 60 patients, corresponding to 5.6% of all patients and 34.9% of patients with SSI. Meshes were removed in total in 42 patients (3.9%) and partially in 18 patients (1.7%). In the laparoscopy and laparotomy subgroups, mesh removal was performed in 7 patients (1.7%) and 53 patients (8.1%), respectively. In the 23 patients with open abdomen treatment, three meshes were removed (13.0%). The number of mesh removals in relation to the type of surgery is outlined in Table 2.

### Independent predictors for SSI

Predictors for SSI in the overall cohort are shown in Table 3. Multivariable regression analysis adjusting for patient-related factors revealed emergency hospitalization, previous laparotomy, duration of operation, laparotomy, as well as bariatric, colorectal, and emergency surgery, higher wound class, and non-polypropylene mesh as independent predictors for SSI in the overall cohort. Hernia surgery was independently associated with a lower risk for SSI.

**Table 3:** Association of disease- and procedure-related factors and surgical site infections in patients undergoing abdominal surgery with intra-abdominal mesh implantation

Overall cohort						
	Univariable analysis			Multivariable analysis^a^		
	OR	95% CI	p-value	OR	95% CI	p-value
Emergency hospitalization	2.257	1.582- 3.220	< 0.001	1.787	1.180–2.708	0.006
Previous laparotomy	1.719	1.087–2.720	0.021	1.745	1.058–2.878	0.029
Duration of operation (hours)	1.187	1.089–1.295	< 0.001	1.193	1.085–1.311	< 0.001
Laparotomy	6.753	4.073–11.196	< 0.001	6.167	3.621–10.504	< 0.001
Type of surgery						
Upper-GI surgery	0.319	0.076–1.344	0.120	0.375	0.085–1.653	0.195
Bariatric surgery	4.956	2.620–9.375	< 0.001	4.641	2.079–10.359	< 0.001
Hernia surgery	0.153	0.100–0.234	< 0.001	0.165	0.104–0.263	< 0.001
Hepato-biliary surgery	1.558	0.856–2.837	0.147	1.586	0.806–3.121	0.182
Pancreatic surgery	1.555	0.566–4.273	0.392	1.704	0.568–5.112	0.342
Colorectal surgery	2.116	1.463–3.060	< 0.001	1.941	1.292–2.916	0.001
Emergency Surgery	3.223	2.127–4.883	< 0.001	2.510	1.554–4.055	< 0.001
Open abdomen treatment	3.494	1.488–8.207	0.004	2.359	0.919–6.055	0.074
Wound class (1 = reference)						
2	4.105	2.546–6.617	< 0.001	4.336	2.614–7.192	< 0.001
3	8.495	4.647–15.528	< 0.001	7.585	3.913–14.703	< 0.001
4	10.153	6.194–16.642	< 0.001	9.408	5.232–16.918	< 0.001
Wound class ≥ 3	4.728	3.351–6.672	< 0.001	3.878	2.574–5.843	< 0.001
Non-polypropylene mesh	1.587	1.114–2.263	0.011	1.818	1.234–2.679	0.003

Uni- and multivariable logistic regression analyses in imputed dataset

*OR* odds ratio, *CI* confidence interval, *SSI* Surgical site infection

^a^Adjusted for sex, age, Body Mass Index, and American Society of Anesthesiologists (ASA) Physical Status Classification System score

Table 4 shows the predictors for SSI in the laparoscopy and laparotomy subgroup. Emergency surgery procedures, bariatric surgery, and higher wound class were identified as independent predictors for SSI in both groups, whereas a significant association of non-polypropylene based meshes with SSI was only found in the laparotomy subgroup, but not the laparoscopy subgroup. Hernia surgery was found to be independently associated with a lower risk for SSI in both groups.

**Table 4:** Association of disease- and procedure-related factors for surgical site infections in patients undergoing abdominal surgery with intra-abdominal mesh implantation

	Laparotomy						Laparoscopy					
	Univariable analysis			Multivariable analysis^a^			Univariable analysis			Multivariable analysis^a, b^		
	OR	95% CI	p-value	OR	95% CI	p-value	OR	95% CI	p-value	OR	95% CI	p-value
Emergency hosp.	1.653	1.121- 2.438	0.011	1.549	0.974–2.462	0.064	3.066	0.985–9.540	0.053	3.115	1.062–9.138	0.039
Previous laparotomy	1.150	0.666–1.986	0.615	1.298	0.709–2.377	0.397	1.392	0.445–4.351	0.569	1.209	0.359–4.075	0.759
Duration of operation (h)	1.067	0.970–1.173	0.183	1.097	0.990–1.215	0.077	1.305	0.973–1.751	0.076	1.225	0.902–1.665	0.194
Type of surgery												
Upper-GI surgery	0.215	0.051–0.912	0.037	0.262	0.059–1.157	0.077	-^c^	–^c^	–^c^	–^c^	–^c^	–^c^
Bariatric surgery	4.037	1.966–8.291	< 0.001	2.682	1.105–6.511	0.029	6.964	1.339–36.229	0.021	7.317	1.051- 50.924	0.044
Hernia surgery	0.291	0.177–0.477	< 0.001	0.294	0.172–0.502	< 0.001	0.136	0.051–0.364	< 0.001	0.138	0.048–0.391	< 0.001
Hepato-biliary surgery	1.249	0.670–2.329	0.484	1.423	0.698–2.900	0.332	–^c^	–^c^	–^c^	–^c^	–^c^	–^c^
Pancreatic surgery	1.021	0.368–2.835	0.968	1.330	0.435–4.062	0.617	–^c^	–^c^	–^c^	–^c^	–^c^	–^c^
Colorectal surgery	1.363	0.924–2.013	0.119	1.356	0.883–2.084	0.165	1.170	0.148–9.263	0.882	1.005	0.123- 8.227	0.996
Emergency Surgery	2.174	1.360–3.474	< 0.001	2.104	1.216–3.641	0.008	12.433	4.496–34.384	< 0.001	14.649	4.384- 48.949	< 0.001
Open abdomen treatment	2.168	0.920–5.111	0.077	1.672	0.650–4.302	0.287	–	–	–	–	–	–
Wound class (1 = ref.)												
2	2.321	1.330–4.050	0.003	2.567	1.424–4.630	0.002	4.313	1.441–12.902	0.009	4.173	1.352- 12.880	0.013
3	5.082	2.599–9.935	< 0.001	4.566	2.204–9.459	< 0.001	–^c^	–^c^	–^c^	–^c^	–^c^	–^c^
4	5.054	2.867–8.909	< 0.001	4.693	2.455–8.972	< 0.001	17.889	4.542–70.461	< 0.001	21.391	4.528- 101.047	< 0.001
Wound class ≥ 3	2.997	2.066–4.347	< 0.001	2.555	1.656–3.942	< 0.001	5.684	1.707–18.928	0.005	5.944	1.573- 22.459	0.009
Non-polypropylene mesh	1.407	0.954–2.075	0.085	1.816	1.183–2.785	0.006	0.785	0.222–2.783	0.708	0.703	0.193- 2.558	0.592

Uni- and multivariable logistic regression analyses in imputed datasets

*OR* odds ratio, *CI* confidence interval, *SSI* Surgical site infection

^a^Adjusted for sex, age, Body Mass Index, and American Society of Anesthesiologists (ASA) Physical Status Classification System score

^b^Adjusted for ASA ≥ 3, as no SSI occurred in ASA = 1 group. ^c^No SSI occurred in investigated group

### Missing data

The proportion of missing values in all data collected was 4.3%. Missing data was found in 257 cases (24.0%) and 15/36 variables (41.7%), respectively. Missing data with regard to individual variables are outlined as denominators in Table 1.

## Discussion

The current study investigated predictors for SSI in patients undergoing IPOM placement for hernia and non-hernia abdominal surgery, including cases with contaminated and infected surgical fields.

The study revealed emergency hospitalization, previous laparotomy, duration of operation, laparotomy, as well as bariatric, colorectal, and emergency surgery, higher wound class, and non-polypropylene meshes as independent predictors for SSI.

To our best knowledge, risk factors for SSI have not been assessed in patients undergoing IPOM implantation in non-hernia abdominal surgery so far. The results support the current practice of IPOM repair for abdominal wall and incisional hernias. On the other hand, when weighting the risks and benefits of IPOM implantation as a prophylactic measure or concomitant hernia repair in abdominal surgery, the knowledge of the above-mentioned risk factors for SSI will help in the decision-making for or against IPOM implantation. In presence of multiple predictors for SSI, implantation of an IPOM should be considered carefully.

At the Inselspital, Bern University Hospital, IPOM implantation is also performed in contaminated and infected surgical fields. This situation allows for an estimation of the impact of the wound class on SSI in patients undergoing IPOM implantation. Initially, mesh implantation was restricted to strictly aseptic conditions, such as in elective abdominal wall hernia repairs. However, prophylactic mesh reinforcement of the abdominal wall, including patients with open abdomen treatment or fascial dehiscence, have challenged these indications [3–5]. Even though this paradigm shift has opened new avenues for the treatment of patients with a high risk for fascial dehiscence and incisional hernias, SSI remain a concern [4, 22]. The results of the current study suggest that mesh implantation should be considered carefully in patients with higher grade abdominal contamination (wound class grade 3 and 4) because of the significantly increased risk for SSI. Adding to the risk of SSI, a contaminated abdomen is typically found in patients undergoing laparotomy for abdominal emergencies, which was identified as an independent predictor for SSI, too. To overcome SSI in presence of IPOM, damage-control surgery principles with delayed abdominal closure may help to reduce the rate of SSI in this group of patients [23]. However, in selected cases, the benefits of IPOM placement, such as definitive abdominal closure, outweigh the risk for SSI and may be the only available treatment option.

In a retrospective cohort study including 103,869 inguinal, umbilical, and ventral hernia operations, mesh explantations due to infection were reported in 6.4%, 29.2%, and 22.4% of patients with superficial, deep, and organ-space SSI, respectively [24]. In the current study, the rate of mesh removal in patients with SSI was higher (34.9%). The higher rate of mesh explantation in the current study may be attributed to the inclusion of partial mesh removals, more frequently performed emergency surgery, and significantly higher proportion of patients with contaminated and dirty-infected surgical fields.

The frequency of wound infections has been reported with a wide range in previous studies [25–28]. Compared to these studies, wound infections were more frequent in the current analysis. This may be well explained by inclusion of patients with other indications for IPOM than abdominal wall hernias, including cases with abdominal contamination and infection.

The current study revealed the use of non-polypropylene meshes as an independent predictor for SSI. As previously reported, polyester-based meshed are associated with mesh infection and mesh-related complications and should not be used in a contaminated environment [29, 30]. Importantly, biologic meshes, which have been developed for contaminated and dirty wounds, seem not to be superior compared to synthetic meshes [31, 32]. In a recent randomized controlled trial, the use of synthetic meshes in a retromuscular position significantly reduced the two-year hernia recurrence risk compared to biologic meshes in patients with contaminated ventral hernias [33]. Considering the results of the current study and previous reports, the usage of non-polypropylene meshes in contaminated or infected surgical fields is not recommended [5].

In line with our observations, the duration of operation has been identified as an important independent predictor for SSI in previous studies [34, 35]. In this context, it should be noted that longer operation times will decrease tissue concentration of antibiotics [36], affect the fatigue of the surgical team, and generally increase the likelihood for a bacteria to contaminate the surgical wound [37]. Therefore, surgeons may not want to prolong an already long-lasting operative procedure by the implantation of an IPOM.

Not surprisingly, the current analysis revealed laparotomy *vs.* laparoscopy as a strong independent predictor for SSI. Regarding SSI, the advantage of laparoscopic interventions compared to open surgery has been shown previously, including in emergency abdominal surgery [38], abdominal wall hernia repair [12, 26], surgery for obese patients [16], patients undergoing colorectal surgery [18], and octogenarians [39]. Thus, to reduce the risk for SSI, IPOM implantation should be performed by a laparoscopic approach whenever possible.

In multivariable analysis, the use of non-polypropylene meshes was significantly associated with SSI in the laparotomy subgroup, but not in the laparoscopy subgroup. This may be explained by the lower sample size and hence reduced statistical power. However, a lower impact of non-polypropylene meshes on SSI in the laparoscopic setting, which is associated with a reduced risk for SSI by itself, should also be taken into account. Interestingly, hernia surgery remained a significant predictor for a lower SSI risk in the laparoscopy subgroup. Thus, in reverse conclusion, laparoscopic IPOM implantation is associated with a higher risk for SSI, if performed prophylactically or in addition to non-hernia surgery.

The strength of this study is the inclusion of patients with IPOM implantation in contaminated and infected surgical fields, as well as the size of the cohort. However, even though SSI were assessed prospectively by the Swiss National Center for Infection Control, the study has several limitations. First, it was not feasible in the scope of this retrospective study to confirm mesh infection in microbiological culture. Second, although multiple variables were assessed in the analysis, potential additional predictors for SSI could have been missed. Third, a moderate amount of missing data was detected in the used dataset. However, missing data were addressed in the analysis by multiple imputation. Based on these limitations, further prospective investigation into the topic is warranted. Future studies should ideally assess mesh infection by microbiological cultures and provide data on the treatment of mesh infections.

## Conclusion

The current study, to our best knowledge, presents the first analysis of predictors for SSI in patients undergoing IPOM placement in hernia and non-hernia abdominal surgery. The study revealed emergency hospitalization, previous laparotomy, duration of operation, laparotomy, as well as bariatric, colorectal, and emergency surgery, higher wound class, and non-polypropylene mesh as independent predictors for SSI. In contrast, hernia surgery was independently associated with a lower risk for SSI. The knowledge of these predictors will help to balance potential benefits of IPOM implantation against the risk for SSI, especially in patients undergoing mesh placement as a prophylactic procedure in abdominal surgery. Such a balanced approach may help to reduce the incidence of SSI in this patient population.

## References

[1] Kokotovic D, Bisgaard T, Helgstrand F (2016). Long-term recurrence and complications associated with elective incisional hernia repair. J Am Med Assoc.

[2] Mathes T, Walgenbach M, Siegel R (2016). Suture versus mesh repair in primary and incisional ventral hernias: a systematic review and meta-analysis. World J Surg.

[3] Jairam AP (2017). Prevention of incisional hernia with prophylactic onlay and sublay mesh reinforcement versus primary suture only in midline laparotomies (PRIMA): 2-year follow-up of a multicentre, double-blind, randomised controlled trial. Lancet.

[4] Jakob MO (2018). Mesh-augmented versus direct abdominal closure in patients undergoing open abdomen treatment. Hernia.

[5] Jakob MO (2018). Prophylactic, synthetic intraperitoneal mesh versus no mesh implantation in patients with fascial dehiscence. J Gastrointest Surg Off J Soci Surg Aliment Tract.

[6] Kohler A (2019). Effectiveness of prophylactic intraperitoneal mesh implantation for prevention of incisional hernia in patients undergoing open abdominal surgery: a randomized clinical trial. JAMA Surg.

[7] Kurmann A, Barnetta C, Candinas D, Beldi G (2013). Implantation of prophylactic nonabsorbable intraperitoneal mesh in patients with peritonitis is safe and feasible. World J Surg.

[8] Plymale MA (2020). Costs and Complications Associated with Infected Mesh for Ventral Hernia Repair. Surg Infect.

[9] Falagas ME, Kasiakou SK (2005). Mesh-related infections after hernia repair surgery. Clin Microbiol Infect.

[10] Mavros MN (2011). Risk factors for mesh-related infections after hernia repair surgery: a meta-analysis of cohort studies. World J Surg.

[11] Centers for Disease Control and Prevention (2023) Surgical Site Infection (SSI) Events. https://www.cdc.gov/nhsn/psc/ssi/index.html

[12] Quiroga-Centeno AC, Quiroga-Centeno CA, Guerrero-Macias S, Navas-Quintero O, Gomez-Ochoa SA (2022). Systematic review and meta-analysis of risk factors for mesh infection following abdominal wall hernia repair surgery. Am J Surg.

[13] van’t Riet, Martijne, PJ de Vos van Steenwijk, H. J. Bonjer, E. W. Steyerberg, and J. Jeekel. (2007). Mesh repair for postoperative wound dehiscence in the presence of infection: is absorbable mesh safer than non-absorbable mesh?. Hernia.

[14] Wilson RB, Farooque Y (2022). Risks and Prevention of Surgical Site Infection After Hernia Mesh Repair and the Predictive Utility of ACS-NSQIP. J Gastrointest Surg Off j Soci Surg Alimentary Tract.

[15] Pierce RA, Spitler JA, Frisella MM, Matthews BD, Brunt LM (2007). Pooled data analysis of laparoscopic vs. open ventral hernia repair: 14 years of patient data accrual. Surg endosc.

[16] Shabanzadeh DM, Sorensen LT (2012). Laparoscopic surgery compared with open surgery decreases surgical site infection in obese patients: a systematic review and meta-analysis. Annals of surg.

[17] Alkaaki A (2019). Surgical site infection following abdominal surgery: a prospective cohort study. Canadian J Surg J Canadien de Chirurgie.

[18] Kulkarni N, Arulampalam T (2020). Laparoscopic surgery reduces the incidence of surgical site infections compared to the open approach for colorectal procedures: a meta-analysis. Techn Coloproctol.

[19] Xu Z (2021). Risk factors for surgical site infection in patients undergoing colorectal surgery: A meta-analysis of observational studies. PLoS ONE.

[20] Swiss National Center for Infection Control (Swissnoso) (2022) https://swissnoso.ch/en/

[21] Horan TC, Gaynes RP, Martone WJ, Jarvis WR, Emori TG (1992). CDC definitions of nosocomial surgical site infections, 1992: a modification of CDC definitions of surgical wound infections. Infect Control Hosp Epidemiol.

[22] Perez-Kohler B, Bayon Y, Bellon JM (2016). Mesh Infection and Hernia Repair: a review. Surg Infect.

[23] Haltmeier T, Falke M, Quaile O, Candinas D, Schnuriger B (2022). Damage-control surgery in patients with nontraumatic abdominal emergencies: a systematic review and meta-analysis. J Trauma Acute Care Surg.

[24] Dipp Ramos R, O'Brien WJ, Gupta K, Itani KMF (2021). Incidence and risk factors for long-term mesh explantation due to infection in more than 100,000 hernia operation patients. J Am College Surg.

[25] Cobb WS, Carbonell AM, Kalbaugh CL, Jones Y, Lokey JS (2009). Infection risk of open placement of intraperitoneal composite mesh. Am Surg.

[26] Kaafarani HM, Kaufman D, Reda D, Itani KM (2010). Predictors of surgical site infection in laparoscopic and open ventral incisional herniorrhaphy. J Surg Res.

[27] Callcut RA (2016). The massive transfusion Score as a decision aid for resuscitation: learning when to turn the massive transfusion protocol on and off. J Trauma Acute Care Surg.

[28] Lavanchy JL, Buff SE, Kohler A, Candinas D, Beldi G (2019). Long-term results of laparoscopic versus open intraperitoneal onlay mesh incisional hernia repair: a propensity score-matched analysis. Surg Endosc.

[29] Leber GE, Garb JL, Alexander AI, Reed WP (1998). Long-term complications associated with prosthetic repair of incisional hernias. Archives Surg.

[30] Majumder A, Petro CC, Liu L, Fayezizadeh M, Novitsky YW (2017). Development of a novel murine model for treatment of infected mesh scenarios. Surg Endosc.

[31] Kockerling F (2018). What is the evidence for the use of biologic or biosynthetic meshes in abdominal wall reconstruction?. Hernia.

[32] Jakob MO, Haltmeier T, Candinas D, Beldi G (2020). Biologic mesh implantation is associated with serious abdominal wall complications in patients undergoing emergency abdominal surgery: a randomized-controlled clinical trial. J Trauma Acute Care Surg.

[33] Rosen MJ (2022). Biologic vs synthetic mesh for single-stage repair of contaminated ventral hernias: a randomized clinical trial. JAMA Surg.

[34] Cheng H (2017). Prolonged operative duration increases risk of surgical site infections: a systematic review. Surg Infect.

[35] Cheng H (2018). Prolonged operative duration is associated with complications: a systematic review and meta-analysis. J Surgl Res.

[36] Tweed C (2005). Prevention of surgical wound infection: prophylactic antibiotics in colorectal surgery. J Wound Care.

[37] Dalstrom DJ (2008). Time-dependent contamination of opened sterile operating-room trays. J Bone Joint Surg Am.

[38] Li Z (2021). Prospective multicenter study on the incidence of surgical site infection after emergency abdominal surgery in China. Sci Rep.

[39] Li Y (2016). Laparoscopic colorectal resection versus open colorectal resection in octogenarians: a systematic review and meta-analysis of safety and efficacy. Tech Coloproctol.

